# Editorial: Diet, nutrition and insect responses to environmental change

**DOI:** 10.3389/finsc.2024.1415809

**Published:** 2024-04-26

**Authors:** C. Ruth Archer, Christopher W. Weldon

**Affiliations:** ^1^ Institute for Evolutionary Ecology and Conservation Genomics, University of Ulm, Ulm, Germany; ^2^ Department of Zoology and Entomology, University of Pretoria, Hatfield, South Africa

**Keywords:** global change, physiology, nutrition, ecology, insects, optimal foraging

## Introduction

**Figure 1:**
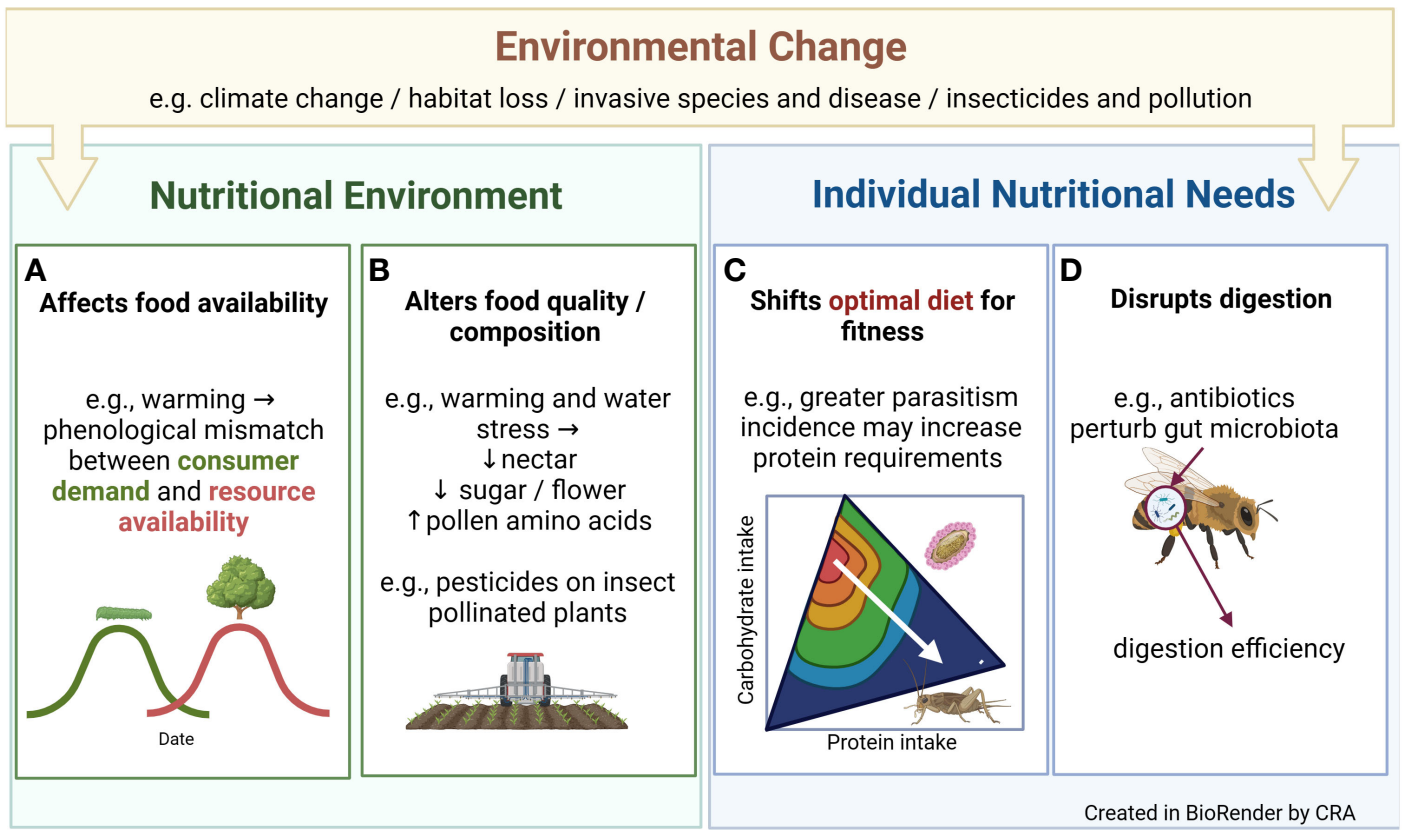
Examples of how environmental change associated with human activity can alter species’ nutritional ecology by affecting food **(A)** availability, **(B)** quality or composition, or altering individual nutritional needs by **(C)** shifting dietary optima or **(D)** disrupting digestion.

An individual’s nutrient intake affects its growth (1), immunity, reproductive success and survival (2). Nutrition influences behaviours including mate choice (3) and migration (4), and interactions between species across trophic levels (5). In sum, nutrition shapes almost all aspects of ecology, from individuals to ecosystems. Understanding a species’ nutritional ecology and physiology therefore helps us understand its broader biology. However, building this understanding is complicated because environmental changes associated with human activity such as climate change or pollution can disrupt species’ nutrition (6) (**Figure 1**). In turn, this can affect the health of individuals, populations and ecosystems. Here, we summarise some routes through which human-driven environmental change can affect species’ nutrition, possible consequences of these effects, and how the manuscripts in this research theme fit within this wider framework.

## A changing nutritional environment

By reducing resource availability or quality, environmental change can make it harder for organisms to consume the nutrients they need. These effects are most obvious in relation to land-use change, for example agricultural intensification has replaced complex ecosystems with monocultures, reducing floral abundance and diversity. More subtly, rising temperatures can alter the seasonal timing of events in species’ life-cycles (i.e., their phenology), potentially desynchronising the appearance of consumers and resources. Warm winters accelerate winter moth caterpillar (*Operophtera brumata*) development for example, meaning that sometimes caterpillars hatch before the young oak leaves they consume have budded (7) (**Figure 1A**). Global change may not just reduce resource availability - invasive species or human provisioning could provide a novel food source, although how this affects consumers depends on the composition of this new food (6).

Even when food availability is constant, its quality may shift e.g. via exposure to pesticides or other pollutants. Food nutritional value may change too, as in the bee-pollinated *Borago officinalis*, where warming and/water stress reduce nectar volume, floral sugar production, and alter pollen polypeptides (8) (**Figure 1B**).

In this research theme, Mubayiwa et al. explored how altered resource quality affects consumer fitness. The authors tested how water stress in different host plants affected fall armyworm *Spodoptera frugiperda* larvae. While larvae reared on suboptimal hosts experienced some reductions in fitness, larvae were relatively robust to variation in host quality. This suggests that using sub-optimal hosts in crop rotations or as intercrops, is unlikely to be an effective tool for tackling this globally invasive species.

## Environmental change alters individual nutritional needs

If the relationship between fitness and nutrient intake is presented as a fitness landscape (**Figure 1C**), environmental change can alter landscape topography – that is, change the nutrients organisms need to consume, to maximise their fitness. For example, increased global connectivity may fuel disease outbreaks (9). This may alter optimal insect diets because nutrition affects immune function, as in decorated crickets (*Gryllodes sigillatus*), where protein consumption helps crickets mount an encapsulation response (2).

Understanding how a changing environment affects dietary optima is a major theme in this research digest. Malod et al. explored how selection on fertility schedules (which may arise due to phenological mismatch between insect herbivores and plant hosts) in the marula fruit fly (*Ceratitis cosyra)* affected associations between protein and carbohydrate consumption, and sex-specific life-history. In this study, fly populations selected for later reproduction had slightly longer lives than control ones and consumed more food, particularly when low in nutrients. Accordingly, a stressful environment with fewer of the carbohydrate-biased resources needed for optimal lifespan and higher female fecundity will likely reduce fitness in this African pest of mango production.

Other articles focused on the relationship between diet and thermal stress resistance.Pullock et al. tested how diet quality affected thermal tolerance in *C. cosyra*. Diet interacted with hardening (a means of resisting thermal stress) to affect resistance to cold, but not warm temperatures. Similarly, Parker and Kingsolver determined how macronutrient intake affected *Pieris rapae* larvae in different temperatures. Diet affected larval life-history, but effects depended on developmental temperature and butterfly population. Both studies agree that the relationship between diet and fitness depends on the thermal environment. Xiao et al. offer insight into how nutrition helps the swallowtail butterfly, *Sericinus montelus*, survive winter diapause and resist thermal stress. The importance of diet is clear given that trehalose acts as a cryoprotectant, and stored resources allow butterflies to survive the metabolic costs of overwintering, which may rise as winters become warmer and diapause is increasingly disrupted.

The environment can also alter individual nutritional needs by affecting digestion. Antibiotics sometimes used by beekeepers to manage pathogens affect how well bees digest protein for example, presumably due to effects on gut microbiota (10) (**Figure 1D**). Disrupting digestion could exacerbate nutritional deficiencies or shift the nutrient blend organisms must consume, to assimilate the nutrients they need. Accordingly, to really understand how a changing environment alters species’ nutritional ecology it is important to understand how the environment affects dietary optima and nutrient regulation at the point of ingestion and digestion, and how this changes over the short and long term (i.e., via plasticity and adaptation).

## Why is this topic important?

Disrupting species’ nutrition could have detrimental effects on those species. In these cases, understanding species’ nutritional ecology in a changing world may help us develop effective conservation strategies. However, some generalists may be robust to shifts in food quality or availability – as demonstrated by Mubayiwa et al. – and the appearance of novel foods may even benefit some consumers. Here, understanding changing nutritional ecology may help us predict species that are likely to become invasive (11) as well as manage existing pests. This idea was explored by Laurie et al. who studied the potential of turmeric as a natural insecticide against the housefly *Musca domestica* and asked if turmeric’s efficacy depended on experimental diet. Results were pronounced – turmeric shortened lifespan in flies independently of their sex and diet.

## Perspective

The articles in this Research Topic shed some light on interactions between the changing environment and species’ nutritional ecology and physiology. Filling the many remaining gaps in our understanding of this topic will help us predict how individuals, populations and food webs respond to drivers of global change.

## Author contributions

CA: Conceptualization, Writing – original draft. CW: Conceptualization, Writing – original draft.

## References

[1] House CM Jensen K Rapkin J Lane S Okada K Hosken DJ. Macronutrient balance mediates the growth of sexually selected weapons but not genitalia in male broad-horned beetles. Func Ecol. (2016) 30:769–79. doi: 10.1111/1365-2435.12567

[2] Rapkin J Jensen K Archer CR House CM Sakaluk SK del Castillo E. The geometry of nutrient space–based life-history trade-offs: sex-specific effects of macronutrient intake on the trade-off between encapsulation ability and reproductive effort in decorated crickets. Am Nat. (2018) 191:452–74. doi: 10.1086/696147 29570407

[3] Stålhandske P. Nuptial gift in the spider *Pisaura mirabilis* maintained by sexual selection. Behav Ecol. (2001) 12:691–7. doi: 10.1093/beheco/12.6.691

[4] Simpson SJ Sword GA Lorch PD Couzin ID. Cannibal crickets on a forced march for protein and salt. Proc Natl Acad Sci USA. (2006) 103:4152–6. doi: 10.1073/pnas.0508915103 PMC1449662 16537500

[5] Kohl KD Coogan SCP Raubenheimer D. Do wild carnivores forage for prey or for nutrients?: Evidence for nutrient-specific foraging in vertebrate predators. BioEssays. (2015) 37:701–9. doi: 10.1002/bies.201400171 25767065

[6] Birnie-Gauvin K Peiman KS Raubenheimer D Cooke SJ. Nutritional physiology and ecology of wildlife in a changing world. Cons Phys. (2017) 5(1):cox030. doi: 10.1093/conphys/cox030 PMC5516125 28740638

[7] Van Dis NE Sieperda G-J Bansal V Van Lith B Wertheim B Visser ME. Phenological mismatch affects individual fitness and population growth in the winter moth. Proc R Soc B. (2023) 290:20230414. doi: 10.1098/rspb.2023.0414 PMC10445013 37608720

[8] Descamps C Quinet M Jacquemart A-L. Climate change–induced stress reduce quantity and alter composition of nectar and pollen from a bee-pollinated species (*Borago officinalis*, Boraginaceae). Front Plant Sci. (2021) 12:3389/fpls.2021.755843. doi: 10.3389/fpls.2021.755843 PMC8542702 34707633

[9] Baker RE Mahmud AS Miller IF Rajeev M Rasambainarivo F Rice BL. Infectious disease in an era of global change. Nat Rev Microbiol. (2022) 20:193–205. doi: 10.1038/s41579-021-00639-z 34646006 PMC8513385

[10] Du Rand EE Stutzer C Human H Pirk CWW Nicolson SW. Antibiotic treatment impairs protein digestion in the honeybee, *Apis mellifera* . Apidologie. (2020) 51:94–106. doi: 10.1007/s13592-019-00718-4

[11] Shik JZ Dussutour A. Nutritional dimensions of invasive success. Trends Ecol Evol. (2020) 35:691–703. doi: 10.1016/j.tree.2020.03.009 32668214

